# Evaluating *cpn60* for high-resolution profiling of the mammalian skin microbiome and detection of phylosymbiosis

**DOI:** 10.1038/s43705-023-00276-y

**Published:** 2023-07-07

**Authors:** Alexander K. Umbach, Champika Fernando, Janet E. Hill, Josh D. Neufeld

**Affiliations:** 1 Department of Biology, University of Waterloo, Waterloo, Ontario Canada; 2 Department of Veterinary Microbiology, University of Saskatchewan, Saskatoon, Saskatchewan Canada

**Keywords:** Microbial ecology, Next-generation sequencing, Microbiome

## Abstract

Despite being the most widely used phylogenetic marker for amplicon-based profiling of microbial communities, limited phylogenetic resolution of the 16S rRNA gene limits its use for studies of host-microbe co-evolution. In contrast, the *cpn60* gene is a universal phylogenetic marker with greater sequence variation capable of species-level resolution. This research compared mammalian skin microbial profiles generated from *cpn60* and 16S rRNA gene sequencing approaches, testing for patterns of phylosymbiosis that suggest co-evolutionary host-microbe associations. An ~560 bp fragment of the *cpn60* gene was amplified with universal primers and subjected to high-throughput sequencing. Taxonomic classification of *cpn60* sequences was completed using a naïve-Bayesian QIIME2 classifier created for this project, trained with an NCBI-supplemented curated *cpn60* database (cpnDB_nr). The *cpn60* dataset was then compared to published 16S rRNA gene amplicon data. Beta diversity comparisons of microbial community profiles generated with *cpn60* and 16S rRNA gene amplicons were not significantly different, based on Procrustes analysis of Bray-Curtis and UniFrac distances. Despite similar relationships among skin microbial profiles, improved phylogenetic resolution provided by the *cpn60* gene sequencing permitted observations of phylosymbiosis between microbial community profiles and their mammalian hosts that were not previously observed with 16S rRNA gene profiles. Subsequent investigation of *Staphylococcaceae* taxa using the *cpn60* gene showed increased phylogenetic resolution compared the 16S rRNA gene profiles, revealing potential co-evolutionary host-microbe associations. Overall, our results demonstrate that 16S rRNA and *cpn60* marker genes generate comparable microbial community composition patterns while *cpn60* better facilitates analyses, such as phylosymbiosis, that require increased phylogenetic resolution.

## Introduction

Mammalian skin microbial communities have a direct influence on host health and disease and share a longstanding evolutionary history with their respective hosts. Initial predatory and nutritional interactions between ancestors of modern bacteria and eukaryotes are hypothesized to have led to multicellularity [1], further developing to include complex metabolic symbioses [2] and vertebrate innate immune responses [3, 4]. Given variations in physiology [5], hair and fur coverage [5, 6], geographic origin and habitat features [7], and evolutionary history and relatedness [8], the mammalian skin environment promotes instances of host-specific microbial co-evolution. Because of niche heterogeneity conferred by mammalian hosts, skin microbial community assemblage is thought to be deterministic (i.e., influenced by specific environmental or host factors) rather than stochastic (i.e., random assemblage and birth-death events) [9]. For specific mammalian orders, microbial evidence demonstrates that host phylogeny correlates with microbial community composition, manifested as “phylosymbiosis” [8].

Phylosymbiosis is a pattern wherein the microbial community composition of a host reflects the host’s environmental and phylogenetic history [10–12], with more distantly related host species showing greater differences in microbial community composition compared to those that are more closely related [8]. Initial microbial community assemblage from either stochastic or deterministic processes could foster close interactions between the host and associated microbiota over time, potentially leading to co-evolutionary relationships and increased associations [12]. Using the 16S rRNA gene phylogenetic marker, Ross et al. showed first evidence of phylosymbiosis within the *Perissodactyla* and *Artiodactyla* orders, which constitute odd-toed and even-toed ungulates, respectively, [8]. Their study also identified a core microbiome common to all sampled orders, represented by soil-associated bacteria, such as *Agrobacterium* and *Arthrobacter*, and taxa from the common skin bacterial genus *Staphylococcus* [8, 13, 14], among others. Within *Primates*, a core axillary microbiome containing *Staphylococcus* was identified as a dominant contributor to microbial beta diversity [5].

Microbiome studies, such as those mentioned above, most often use short-read amplicon 16S rRNA gene sequencing because of the costs associated with full-length 16S rRNA gene sequencing and metagenomics. However, limited phylogenetic “resolution” of the 16S rRNA gene, when used for short-read sequencing, results in sequence classification mostly to the genus-level [15], preventing detailed investigations of microbial species and specific populations, such as *Staphylococcaceae*. This limitation to phylogenetic resolution may also mask observations of phylosymbiosis perhaps only present at lower taxonomic levels. Investigating the mammalian skin microbiome using alternative gene markers with higher phylogenetic resolution, such as *cpn60* [16], could reveal key microbe-host associations that may indicate existing or potential future co-evolutionary relationships within the class *Mammalia*, with implications for mammalian skin health and disease.

Several features of the *cpn60* gene, alternatively known as *hsp60* and *groEL*, make it useful for short-read amplicon microbial community profiling studies. The *cpn60* gene is universal, encoding for a 60 kDa GroEL protein belonging to a family of type I chaperonins present in bacteria and chloroplasts, with equivalent type II chaperonins found in archaea and eukaryotes. Like the 16S rRNA gene, the presence of *cpn60*, and function of GroEL, is essential for cell survival and thus present in all prokaryotic life [17]. However, unlike the 16S rRNA gene, only a single copy of *cpn60* is present in most prokaryotic genomes [16]. Additionally, the *cpn60* gene contains a much greater sequence diversity compared to the 16S rRNA gene variable regions used in short-read amplicon studies, with a higher diversity in nucleotide identity and larger differences in sequence similarity between species [16]. Furthermore, the primers used to amplify the 274–828 “universal target” region of the *cpn60* gene [16] produces an amplicon of ~554 bp in length. Although this length exceeds current short-read high-throughput sequencing technology, only 150–250 bases of the forward read is necessary to produce sufficient data for phylogenetic classification to the species level [18], allowing it to be used on current sequencing platforms (e.g., MiSeq, Illumina). Additionally, recent validation of naïve-Bayesian classification using the RDP classifier demonstrated a high degree of species-level classification using *cpn60* reference sequences [19]. Because of intrinsic features of the *cpn60* gene, the continued development of primers [20, 21] and reference databases [22], prior in silico and in situ validation [16, 18, 19], and history of use in both universal and targeted microbial profiling studies [23–29], the *cpn60* gene is a powerful complement to the 16S rRNA gene as a universal phylogenetic marker within the context of microbial community profiling and phylosymbiosis/co-evolution research of the skin microbiome.

By leveraging the increased phylogenetic resolution provided by the *cpn60* gene, in combination with amplicon sequence variants (ASVs) capable of identifying individual amplicon sequences [30], skin microbiota can be more thoroughly profiled and evaluated for patterns such as phylosymbiosis and co-evolution. As well, core microbiota associated with the skin, such as *Staphylococcaceae*, can be profiled at increased phylogenetic resolution similar to multi- or single-locus sequence typing approaches [31], balancing high-specificity and universal detection of bacteria. Although previous studies have used the *cpn60* gene to profile the human vaginal [23–25] and pig feces microbiomes [32], it has yet been applied to profile the broader mammalian skin microbiome. This study uses comparative microbial profiling of paired *cpn60* and 16S rRNA genes datasets to provide additional support for using *cpn60* to profile skin microbiota, confirm previous reports of phylosymbiosis on mammalian skin [8], and present novel insight into specific microbial populations of mammalian skin and their potential interactions with their respective hosts. This study further demonstrates how the *cpn60* database, cpnDB [22], can be integrated into the full microbial sequence data QIIME2 pipeline for producing complete *cpn60* gene amplicon datasets.

## Methods

### Sample selection, PCR amplification, and high-throughput sequencing

Genomic DNA from mammalian skin swabs was extracted as part of a previous 16S rRNA gene survey generated from amplification of the V3-V4 region with the universal prokaryotic Pro341F/Pro805R primers [8]. From these, a representative 95-sample subset was chosen for *cpn60*-based sequencing and microbial profiling. Both PCR amplification and high-throughput sequencing of samples were completed at the University of Saskatchewan as previously described [20, 21]. The *cpn60* gene was amplified by PCR using a primer mix comprised of 100 µM M279 (5′ – GAIIIIGCIGGIGAYGGIACIACIAC – 3′), M280 (5′ – YKIYKITCICCRAAICCIGGIGC– 3′), M1612 (5′ – GAIIIIGCIGGYGACGGYACSACSAC– 3′), and M1613 (5′ – CGRCGRTCRCCGAAGCCSGGIGCCTT– 3′). All primers contained Illumina adapter sequences on the 5′ end. Primers were combined in a 1:3 molar ratio of M279 and M280 (3 µL each) and M1612 and M1613 (9 µL each), then diluted in Ultrapure water to a total volume of 300 µL. All PCR tubes, plates, and Ultrapure water used for PCR and sequencing were decontaminated prior to use by exposure to UV light for 20 min. A PCR master mix was prepared using 38.1 µL of UV-treated Ultrapure water, 0.4 µL of Invitrogen Platinum *Taq* (ThermoFisher Scientific), 5 µL of 10X Thermopol buffer, 1 µL of 10 mM dNTPs, and 1 µL of the 1:3 primer cocktail for a total reaction volume of 50 µL for 2 µL of template. The amplification reaction conditions were 95 °C initial denaturation for 5 min, followed by 40 cycles of denaturation at 95 °C for 30 s, annealing at 60 °C for 30 s, extension at 72 °C for 30 s, and a final extension at 72 °C for 2 min. All PCR amplifications were first visualized on a 1% ethidium bromide gel then extracted from the gel and purified using NucleoMag beads (Macherey-Nagel) as previously described [21]. The purified amplicon library was sequenced using a 401 × 101 cycle using TG MiSeq Reagent Nano Kit v2 (Illumina Canada, MS-103-1003) on a MiSeq System (Illumina).

### Processing of sequence reads

Demultiplexed sequences were used to generate ASVs using QIIME2 version 2019.10.0 [33, 34]. Only forward reads containing a 400-nucleotide long amplicon were imported into QIIME2 and truncated to 200 nucleotides using DADA2 version 2019.10.0 [35]. The trimmed reads were then denoised and chimeric sequences removed prior to ASV generation using DADA2. A QIIME2 naïve-Bayes taxonomy classifier [33, 34] was constructed using a combination of reference sequences from the cpnDB reference database [22] and NCBI [36]. Representative sequences produced from DADA2 were used with nBLAST to query the NCBI non-redundant, cultured-only reference database, incorporating an e-value threshold of 1e^−6^, using Entrez Direct [37]. The top three results for each sequence query were collected to create a database containing 16,624 sequences. Replicate entries and sequences below 180 nucleotides were removed (4400) and the remaining sequences (12,224) were combined with the cpnDB_nr (5489) to create a final database of 17,713 *cpn60* gene reference sequences. Taxonomic lineages for the representative sequences were obtained from NCBI using the reference sequence accession numbers and subsequently formatted to integrate within the QIIME2 environment.

All *cpn60* sequences generated for the current study were deposited in the European Nucleotide Archive (ENA) under project accession number PRJEB43503. A *cpn60* ASV table has been made available at https://figshare.com/articles/dataset/cpn60_ASV_table/14955753.

### Mammalian COXI gene, 16S rRNA gene, and *cpn*60 gene microbial dendrograms

Cytochrome oxidase I (COXI) genes for the Cape eland, donkey, goat, horse, olive baboon, Przewalski’s horse, sheep, spotted hyena, and Sumatran orangutan were obtained from a previous study [8] and used to construct a mammalian COXI-based phylogeny. The mammalian COXI gene sequences were aligned using ClustalW [38] in MEGA X version 10.1.8 [39], with trimming and gap removal as appropriate. The optimal nucleotide substitution model was determined using JModelTest2 version 2.1.10 [40, 41]. For the mammalian COXI gene, a maximum likelihood dendrogram was constructed using MEGA X with a GTR + G + I substitutional model and with a confidence assessment of 1000 bootstraps. The mammalian phylogeny was then compared with literature and confirmed for accuracy.

Microbial dendrograms based on community composition were generated using 16S rRNA gene sequences obtained from a previously generated amplicon dataset [8] and the *cpn*60 gene sequences from this current study. For the 16S rRNA and *cpn60* gene microbial dendrograms, the respective ASV tables were sample-collapsed based on mammalian host, and the ASV read counts were summed for each category. Bray-Curtis, unweighted UniFrac, and weighted UniFrac distance matrices for the ASV tables were generated using the QIIME2 *diversity beta-rarefaction* command, rarefied to 1000 reads, and used to construct UPGMA dendrograms with a confidence assessment of 1000 bootstraps.

### Procrustes and phylosymbiosis

To compare microbial community composition between 16S rRNA and *cpn60* gene datasets, Procrustes analyses were performed on principal coordinate analysis (PCoA) ordinations generated using Bray-Curtis and weighted/unweighted UniFrac metrics. Distance matrices were generated using the *qiime2 diversity core-metrics-phylogenetic* command, then exported with *qiime-tools export*. Procrustes analyses were completed in R using the *vegan* package and *protest* command with 100,000 permutations and plotted using *ggplot*.

Mammalian COXI gene and microbial 16S rRNA and *cpn60* gene dendrograms were compared and evaluated for congruence using the *vegan, phangorn*, and *ape* packages in R as previously described [8]. Phylosymbiosis was assessed with Robinson-Foulds scores for both the 16S rRNA and *cpn60* gene dendrograms against the mammalian COXI dendrogram. The significance of the Robinson-Foulds metric was determined by comparing the mammalian COXI gene dendrogram against 100,000 randomly generated trees containing identical terminal nodes (i.e., taxa). Congruency between dendrograms were measured with the normalized Robinson-Foulds score, ranging between 0 and 1, with 0 representing perfect congruency. Random dendrograms were considered significant if they obtained Robinson-Foulds scores equal to or greater than those obtained from comparisons between the mammalian phylogeny and the microbial gene (16S rRNA or *cpn60*) dendrograms.

## Results and discussion

### Mammalian host swab sample selection

**Fig. 1:**
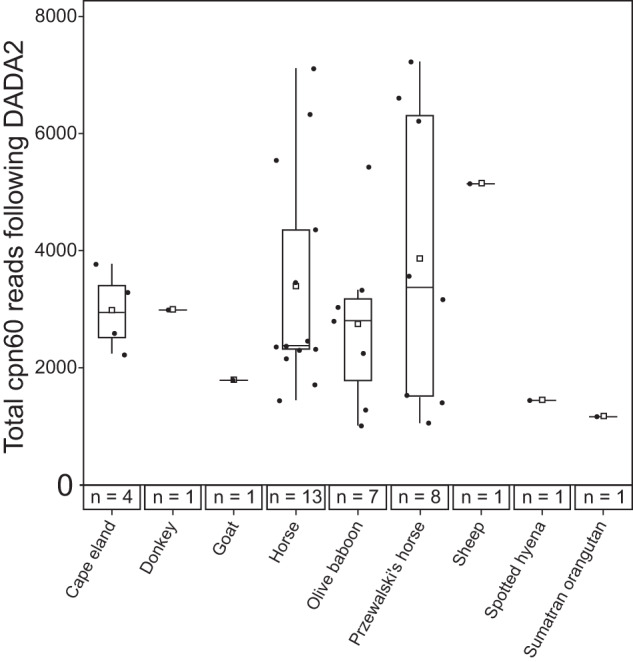
The total number and distribution of *cpn60* gene amplicons within the final 37-sample dataset, following processing through DADA2. The average number of amplicons per mammalian host is indicated with a box.

To investigate mammalian skin microbial communities using the *cpn60* gene, 95 representative skin swab samples, already extracted for genomic DNA and sequenced from a previous project [8], were selected for additional amplification and sequencing of the *cpn60* gene. This *cpn60* gene amplicon dataset contained samples from 19 unique mammals, with varying representation with respect to number of samples and read proportions (Table S1). Of the 95 samples submitted for sequencing, 88 unique samples contained at least one read, with most samples associated with fewer than 500 total reads. The horse and Przewalski’s horse samples represented 36.6% and 25.4% of all sequenced *cpn60* gene reads, respectively, followed by the olive baboon (15.8%) and Cape eland (10.0%). All other mammalian host groups contained reads representing less than 5% of total *cpn60* gene reads. To avoid downstream analysis issues related to shallow sampling depths, all samples with fewer than 1000 reads were removed, resulting in a final dataset of 37 samples (Fig. 1). This subset contributed 97.6% (118,645) of all *cpn60* gene reads (121,622). Although overall read loss was minimal, mammalian host representation was reduced from 19 to 9: the Cape eland, donkey, goat, horse, olive baboon, Przewalski’s horse, sheep, potted hyena, and Sumatran orangutan (Fig. 1).

The loss of mammalian host representation due to shallow sequencing depth was unexpected and might have methodological cause. Microbial biomass on mammalian skin is variable and can be comparably low, with dry-swab sampling of the skin producing the least amount of biomass compared to other methods [42]. The samples used in this current study were collected via the dry-swabbing method [8] and therefore are likely to have relatively low biomass and associated DNA yields. Additionally, the samples were several years old (i.e., four years at time of sequencing) and stored at −20 °C instead of the recommended −80 °C for skin swab samples [42]. Combined, these factors may have affected the integrity of the genomic DNA and associated *cpn60* templates, and subsequent sequencing depth. The *cpn60* gene itself has a median copy number of one per genome [16] and thus samples could be more susceptible to DNA degradation impacting target amplification. Future investigations of specific communities of the mammalian skin microbiome using the *cpn60* gene would benefit from using genomic DNA that has been recently extracted from mammalian skin samples, ideally using wet-swabbing or tape-stripping to increase biomass collection [42].

### Comparing taxonomic profiles and community composition of 16S rRNA and *cpn60* gene amplicon datasets

**Fig. 2:**
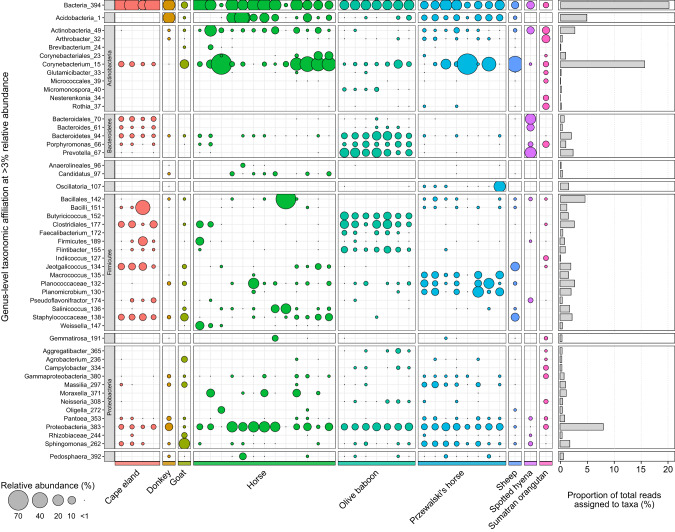
The distribution, relative abundance, and taxonomic affiliation of *cpn60* gene amplicons sequenced from the skin of mammalian hosts. Generated ASVs were collapsed to the genus level and filtered at >3% relative abundance. Bubble sizes represent the relative abundances of taxa in each sample. Taxa unresolved to a genus level were labeled according to their next resolved taxonomic level.

Despite a reduction in host representation, *cpn60*-based sequencing provided valuable insight into the mammalian skin microbiome. The olive baboon samples contained distinct and comparatively uniform microbial profiles (Fig. 2). These profiles were represented by *Prevotella*, *Prophyromonas*, and *Butyricicoccus*, which were most abundant in the olive baboon samples. The Cape eland, horse, and Przewalski’s horse samples had less consistent microbial profiles among samples. The Cape eland samples contained sequences associated with *Jeotgalicoccus* that were shared among the goat, horse, and sheep samples. For the horse samples, microbial profiles were variable among samples and contained unique horse-associated sequences associated with *Moraxella*. The microbial profiles of the Przewalski’s horse samples were more uniform and contained sequences affiliated with *Planomicrobium* and *Macrococcus;* these taxa were nearly absent in other mammalian host microbial profiles. Of mammalian hosts with single sample representation, the Sumatran orangutan microbial profiles included more unique genera (nine) than the donkey (four), goat (two), sheep (zero), and spotted hyena (one). Across all samples, *Corynebacterium*-associated sequences dominated and were represented in moderate relative abundance (>5%) within the goat, horse, olive baboon, Przewalski’s horse, and sheep, although in some cases represented as much as 75% of total community. Sequences affiliated with *Acidobacteria* were also present on the donkey, horse, olive baboon, and Przewalski’s horse at relative abundances ranging from 4 to 38%. The most observed sequences belonged to unclassified bacteria (*Bacteria*_394), which were present in 35 of the 37 samples in high abundance, as well as an unresolved *Proteobacteria* (*Proteobacteria*_383) in 30 of the 37 samples.

**Fig. 3:**
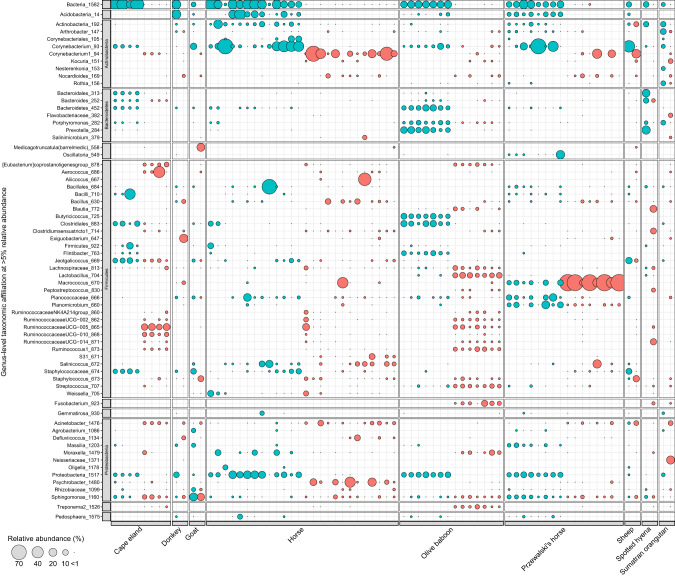
Comparison of the distribution, relative abundance, and taxonomic affiliation of the 16S rRNA (red bubbles) and *cpn60* (blue bubbles) gene amplicon datasets. The ASVs were collapsed to the genus level and filtered at >5% relative abundance. Bubble sizes represent the relative abundances of taxa in each sample. Taxa unresolved to a genus level were labeled according to their next resolved taxonomic level.

Comparisons against the previously classified 16S rRNA gene dataset showed inconsistencies among taxonomic profiles (Fig. 3, Table S2). Although different gene markers will be subject to various biases such as gene copy number [43, 44], nucleotide GC content [45, 46], the region targeted and primers used for amplification [47, 48] and certain bacterial proportions [49], the primary cause for dissimilarity in taxonomic profiles is the separate reference database used for each dataset. The availability of curated *cpn60* gene reference databases is limited. The only currently maintained *cpn60* database is the cpnDB [22], which at the timing of this study contained ~7000 sequences in its non-redundant database. Although this was increased to 17,713 sequences by combining it with nucleotide BLAST results, the cpnDB is far surpassed by the SILVA 138.1 16S rRNA gene database, which contains over 510,000 non-redundant reference sequences [50]. A large proportion of the *cpn60* gene reads were unclassified bacteria or remained unresolved to the genus level compared to the 16S rRNA gene data (Table S2). An increase in *cpn60* gene database coverage would improve classification and facilitate direct comparisons of taxonomy between microbial profiles generated from separate phylogenetic markers.

In addition to limited database coverage, direct comparisons of taxonomic profiles between two phylogenetic markers requires that ASVs be collapsed into taxonomic levels (e.g., genus or species), which is dependent on the taxonomic structuring of the database itself. Much how ASVs with a single nucleotide difference may be interpreted as a unique ASV, taxonomic lineages that differ by as little as one character will be classified as separate taxa within QIIME2. In this study, the 16S rRNA gene dataset was assigned taxonomy using the SILVA database [50, 51], which obtains its taxonomic information from a combination of sources, including the NCBI and GTDB [52], and undergoes further manual annotation. In contrast, the cpnDB does not currently maintain a taxonomy reference database. Instead, the *cpn60* gene dataset was assigned taxonomy based on an NCBI-derived taxonomy database generated for this study as a requirement for implementation into the QIIME2. As such, differences between the *cpn60* and 16S rRNA gene profiles may have been affected by incongruous taxonomies between databases and may not accurately reflect true differences. For example, two *Corynebacterium* classifications (i.e., Corynebacterium_93, and Corynebacterium1_94) were present in the data (Fig. 3). A successful collapse to the genus level should have placed the associated ASVs into a single common *Corynebacterium* genus. In this case, the difference is the inclusion of a “1” at the end the *Corynebacterium* lineage within the 16S rRNA gene taxonomy file. Similarly, *Massilia* is separated into *Massilia*_1203 and *Massilia*_1347 (data not shown). Here, the differences are a result of higher taxonomic classification: *Massilia* has been reclassified under class *Gammaproteobacteria* within the 16S rRNA taxonomy based on the GTDB [52], whereas the NCBI taxonomy and SILVA database currently maintains the original class *Betaproteobacteria* lineage. As such, ASVs associated with the same genus are listed as two separate taxa instead of one, and the difference is “invisible” at the genus level. Should the *cpn60* gene continue to be used as an alternative phylogenetic marker to the 16S rRNA gene, as well as implemented within the QIIME2 environment, it is important that a reference taxonomy database containing compatible taxonomic lineages with the SILVA database (or other routinely accessed reference databases) be maintained alongside the cpnDB so that direct comparisons can be made. Alternatively, a combination of BLAST and Smith-Waterman alignments (watered-BLAST) have been used previously to assign taxonomy to *cpn60* gene datasets [25], although this method also uses the NCBI taxonomy database and would be subject to the same taxonomic incompatibility issues.

**Fig. 4:**
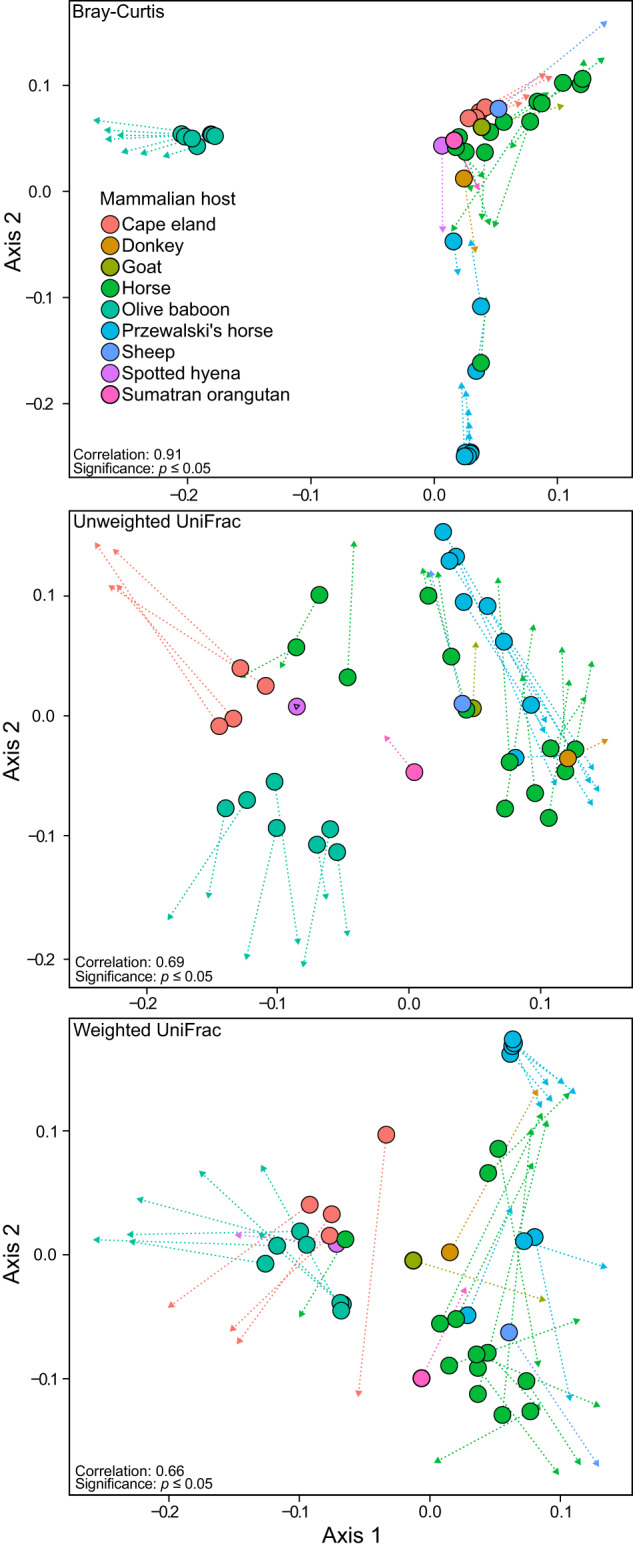
Procrustes analyses of PCoA ordinations produced by the 16S rRNA (primary plot; circles) and *cpn60* (secondary plot; triangles) gene amplicon datasets. Triangles (arrow-heads) and connecting lines indicate the change in ordination space of samples between datasets. The analyses were completed with 100,000 permutations to calculate significance.

Microbiome data generated from two separate universal prokaryotic phylogenetic markers should, ideally, produce similar compositional profiles, despite any difference in taxonomic classification. PCoA and Procrustes analyses were used to compare microbial community composition dissimilarity between *cpn60* and 16S rRNA gene datasets, independent of taxonomic classifications and bias imparted by chosen reference databases. The ordinations produced from both datasets showed significant correlations (*p* < 0.05), with correlation coefficients of 0.91, 0.69, and 0.66 for Bray-Curtis, weighted UniFrac, and unweighted UniFrac, respectively (Fig. 4). The olive baboon sample compositions were distinct, with samples grouping separate from other mammalian hosts for each tested metric. The Przewalski’s horse and Cape eland samples also grouped with their respective hosts, and all other samples grouped homogenously among mammalian hosts for each tested diversity metric.

Because Bray-Curtis only accounts for presence and abundance data, a high correlation of the Bray–Curtis metric indicates that both the *cpn60* and 16S rRNA gene datasets contain a similar proportion and distribution of taxa within their respective samples. Including phylogenetic data (i.e., UniFrac) can unmask potential differences with respect to ASV relatedness and provide insight into whether microbial community composition between the *cpn60* and 16S rRNA gene datasets are comparable. Indeed, weaker correlations observed using the UniFrac metrics could indicate that the *cpn60* gene amplicon provides additional phylogenetic information that the V3-V4 region of the 16S rRNA gene cannot. As the V3-V4 fragment of the 16S rRNA gene often lacks sufficient nucleotide diversity to confidently resolve species [15], diversity metrics dependent on phylogenetic resolution will be similarly limited. In contrast, the *cpn60* gene amplicons contain enough phylogenetic information to resolve species and radiate underlying phylogenetic trees [18, 53–56]. Additional validations using a subset of this current dataset support that *cpn60* gene amplicons more clearly resolves species-level taxa compared to the 16S rRNA gene amplicons (Fig. S1). The differences in resolution will influence dissimilarity measurements resulting in changes in observed sample similarity, and ultimately differences in Procrustes correlation between datasets. Specifically for the unweighted UniFrac metric, the phylogenetic resolution provided by the *cpn60* gene would have considerable impact because resolving of shallow-branch taxa contribute nearly 90% of the sample distance [53], resulting in lower correlations. For the weighted UniFrac, deep-branch taxa are largely responsible for sample distances [53] and therefore should be less influenced by an increase in phylogenetic resolution unless it results in changes to deep-branch topology (i.e., phylum or class-level changes). Nonetheless, all Procrustes tests (Fig. 4) indicate microbial community compositions generated from *cpn60* and 16S rRNA gene datasets are significantly similar to each other and can be used to make similar conclusions regarding the mammalian host-associated microbiome and phylosymbiosis.

### Phylosymbiosis and mammalian host–microbe associations

**Fig. 5:**
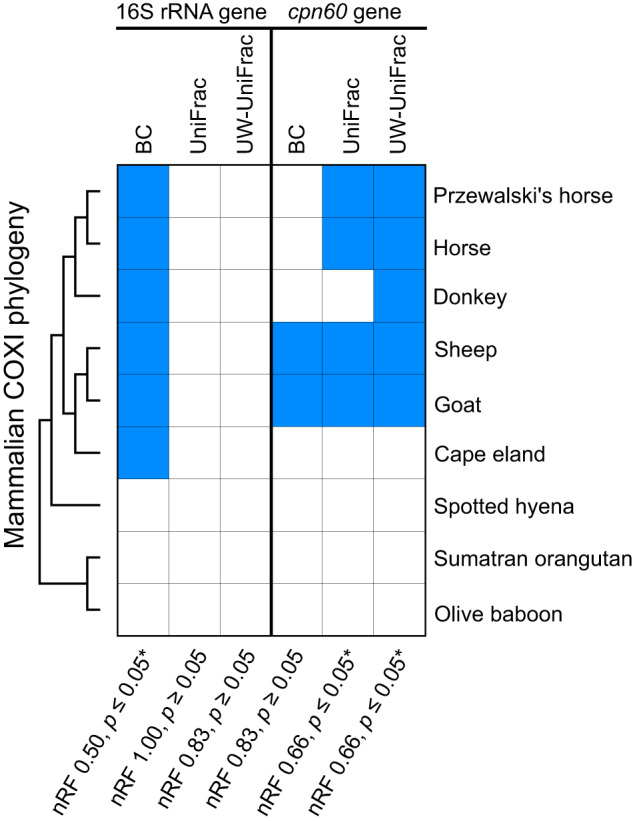
Assessment of phylosymbiosis by comparing a mammalian COXI phylogeny with 16S rRNA and *cpn60* gene microbial dendrograms generated from Bray–Curtis (BC), weighted UniFrac (UniFrac), and unweighted UniFrac (UW-UniFrac) analyses. Blue squares indicate identical clades between the mammalian phylogeny and microbial dendrogram. Congruency was tested for significance using a normalized Robinson-Foulds measure (nRF) which ranges between 0 and 1, with 0 representing perfect congruity. Significant observations of tree congruity are indicated with an “*”.

The phylogenetic resolution provided by the *cpn60* marker gene should allow for additional observations of host-microbe associations, particularly if those associations are at finer taxonomic levels. Patterns of phylosymbiosis within *cpn60* and 16 S rRNA gene amplicon microbial profiles were assessed by comparing microbial community composition dendrograms against a COXI mammalian dendrogram representing mammalian phylogenetic history. Significant (*p* = 6.73 × 10^−3^) patterns of phylosymbiosis were observed in the 16S rRNA gene Bray–Curtis microbial dendrogram for clades containing the Cape eland, goat, and sheep (*Artiodactyla*) and the donkey, Przewalski’s horse, and horse (*Perissodactyla*), although these observations were not significant for the *cpn60*-based microbial dendrogram (Fig. 5). In contrast, significant results were observed for the *cpn60* gene amplicon unweighted (*p* = 4.36 × 10^−2^) and weighted (*p* = 4.43 × 10^−2^) UniFrac microbial dendrograms for *Artiodactyla* (Cape eland excluded) and *Perissodactyla*, but not within the 16S rRNA gene-based dendrograms. Evidence for phylosymbiosis was absent for the *Primates* (olive baboon and Sumatran orangutan) and *Carnivora* (spotted hyena). These observations further confirm that microbial communities of *Perissodactyla* and *Artiodactyla* are influenced by host evolutionary history, as observed previously using the 16S rRNA gene [8]. Because the mammalian hosts included in the study varied in location and age, and are potentially more influenced by “environmental” microorganisms, phylosymbiosis patterns could be masked when mammals from multiple mammalian orders are included together for analysis [8]. This current study relies on samples originally obtained from the same publication, thus similar confounding factors may also influence the current observations, and have been previously addressed [8]. Nonetheless, *cpn60*-based microbial dendrograms produced significant and congruent phylosymbiosis results for *Artiodactyla* and *Perissodactyla* using UniFrac measures, without requiring isolating them from other mammalian orders, as performed previously [8]. That phylogenetic-based UniFrac metrics produced significant phylosymbiosis results only with the *cpn60-*based microbial profiles suggests that the phylogenetic resolution provided by the *cpn60* gene reveals subtle compositional differences and unmasks phylosymbiosis patterns not observed in 16S rRNA gene dataset. However, mammalian host representation is limited within this current study, and the inclusion of additional samples might result in re-masking of phylosymbiosis patterns.

### Using *cpn60* to investigate specific microbial populations and their host associations

**Fig. 6:**
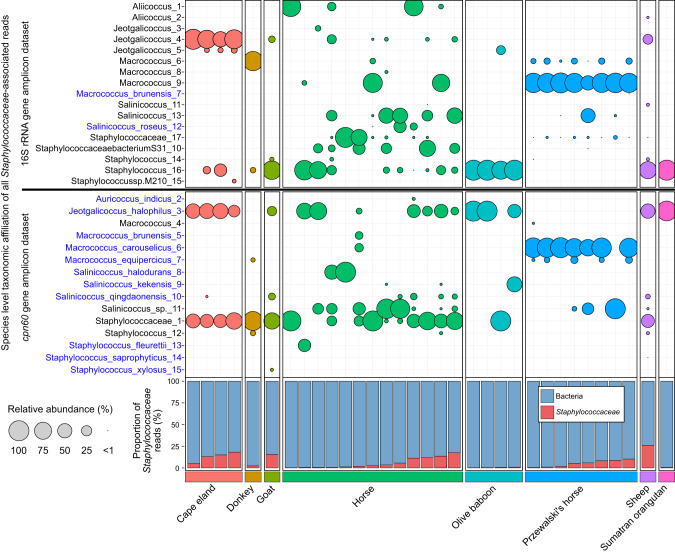
Relative abundance and distribution of *Staphylococcaceae* species among mammalian hosts within the *cpn60* and 16S rRNA gene amplicon datasets. Taxa resolved to a species level are indicated in blue. Bubble sizes represent the relative abundances of specific taxa in each sample, calculated from the total number of *Staphylococcaceae* reads. The proportion of *Staphylococcaceae* reads in the *cpn60* gene amplicon dataset is indicated for each sample within the bottom bar chart.

A proposed benefit of using the *cpn60* gene for microbiome profiling is the ability to universally detect and resolve specific microbial populations and their associations with an environment or host. To test this advantage, family *Staphylococcaceae* was selected for additional analysis, given the ubiquity of affiliated taxa among mammalian hosts [8] and the relevance of certain members to skin health and disease. Of the 37 samples, 33 (89%) contained *Staphylococcaceae-*associated reads. The proportion of reads associated with *Staphylococcaceae* varied among mammalian hosts and ranged from 0.08% (2/2307 reads) to 26.0% (1339/5,142 reads), with most samples (23/33, 69.9%) below 10% relative abundance (Fig. 6). The spotted hyena sample contained no *Staphylococcaceae*-associated reads and was therefore removed from further analysis. The olive baboon and Sumatran orangutan samples contained the fewest number of reads associated with *Staphylococcaceae*, with no sample exceeding 1% relative abundance. The distribution of specific *Staphylococcaceae* species varied among mammalian hosts. Reads associated with *Jeotgalicoccus halophilus* were present among all mammalian hosts excluding the donkey and Przewalski’s horse, and unresolved *Staphylococcaceae* species were similarly present, though absent from the Przewalski’s horse and Sumatran orangutan. For the Przewalski’s horse samples, *Macrococcus carouselicus* was the predominant *Staphylococcaceae* species in most samples, followed by *Macrococcus equipercicus*, which was absent from the horse samples. The horse and Przewalski’s horse samples both contained *Salinicoccus* species, but otherwise had minimal overlap between hosts. The horse samples were the most variable with overlap between many other mammalian hosts, although contained sequences uniquely associated with *Staphylococcus fleurettii*, *Salinicoccus halodurans*, and *Macrococcus brunensis*. The Cape eland samples contained *Staphylococcaceae* populations that evenly split between an unresolved *Staphylococcaceae* and *Jeotgalicoccus halophilus*.

Comparisons made between the *cpn60* and 16S rRNA gene amplicon *Staphylococcaceae* profiles show improved species classification of *Staphylococcaceae* populations when using the *cpn60* gene (Fig. 6, Fig. S1). Most of the profiles produced using the 16S rRNA gene contained large relative abundances of unresolved *Staphylococcaceae* sequences, with only two resolved species present in the dataset (i.e., *Macrococcus brunensis* and *Salinicoccus roseus*). In comparison, the *cpn60* gene profiles contained mainly species-resolved populations, with few exceptions. The benefit of improved phylogenetic resolution is most evident for Prezwalski’s horse samples, wherein most *Staphylococcaceae*-associated sequences resolved to *Macrococcus carouselicus* or *Macrococcus equipercicus* between the 16S rRNA gene and *cpn60* gene dataset. The distribution and relative abundances of these taxa, in combination with previous observations of phylosymbiosis in *Perissodactyla*, suggests a host-specificity for Przewalski’s horses and potential co-evolutionary influence. The Przewalski’s horse is considered a true “wild” horse, having remained undomesticated and relatively isolated to the Asian steppes [57], although these current samples are sourced from the Toronto zoo (Ontario, Canada). The skin environment of Przewalski’s horses could differ from the common domesticated horses include in this study, and thus might influence microbial community assemblage. Importantly, although the horses included in this analysis were regularly brushed (i.e., daily to weekly), the Przewalski’s horses were left ungroomed. As such, the population of *Staphylococcaceae* observed on Przewalski’s horses could represent a more “natural” population, in contrast to domesticated horses where their microbiota is continually disturbed by human interaction. Differences or disruption in *Staphylococcaceae* populations on the Prezewalski’s horse and domesticated horse could impact host susceptibility to disease given that *Staphylococcaceae* has strong associations in the development of equine pastern dermatitis [58].

The most prominent resolved *Staphylococcaceae* representative affiliated with the *cpn60* dataset was *Jeotgalicoccus halophilus*. The comparable 16S rRNA gene dataset [8] also contains the genus *Jeotgalicoccus* with similar proportions for the Cape eland and several horse samples, although most *Staphylococcaceae* affiliated sequences belong to the *Macrococcus* genus (83.7%). None of the *Jeotgalicoccus* associated ASVs within the 16S rRNA gene dataset were resolved to *J. halophilus*. The genus *Jeotgalicoccus* was originally isolated from a traditional Korean fermented seafood [59], with other representatives captured as aerosols from pig [60] and turkey [61] farms. Specifically, *J. halophilus* was first isolated from a salt lake [62] and has since been detected as an airborne bacterium in hatcheries [63] and in association with marine corals [64]. At this writing, there is no additional literature that mentions *J. halophilus* in association with mammalian skin, although this does not exclude their previous detection. Querying the NCBI database returned sequences obtained from environmental studies previously mentioned, but also included a bovine mastitis study in which *J. halophilus* was detected [65].

Although *Jeotgalicoccus* was detected previously on mammalian skin using the 16S rRNA gene [8], the *cpn60* gene has enabled resolution of *J. halophilus* from other species therein. Within the *Primates*, *J. halophilus* represents the totality of the *Staphylococcaceae* associated reads (Fig. 6), although this is due to low *Staphylococcaceae* read depth. However, the Cape eland samples have a considerably higher *Staphylococcaceae* read depth, with *J. halophilus* representing at least half the total reads, and are the predominant species-resolved taxon. Similarly, the sheep sample, which contained the highest proportion of *Staphylococcacea*-associated reads, also had a high proportion of *J. halophilus*, suggesting that the relative abundance and prevalence of this taxon is not an artifact of limited read depth. Additionally, given its absence in several samples and two mammalian hosts (i.e., donkey and Przewalski’s horse) as well as controls, it is unlikely to be a cross-contaminant introduced from within the lab during sample extraction and processing.

The importance or role of *J. halophilus* in the context of mammalian skin is unknown. As a facultative anaerobe with basic metabolic requirements, a growth range of 4–40 °C, and salt tolerability of 0.1 to 16% w/v [62], it is well adapted for survival on mammalian skin. As well, it is coagulase and oxidase positive and resistant to several natural antibiotics [62], which could indicate its ability to act as an opportunistic pathogen. It is difficult to make conclusions about the influence of detected host associated *Staphylococcaceae* with respect to skin function, health, and disease. For example, *Staphylococcus fleuretti*, found in a single horse sample, is a coagulase-negative organism associated with various animal diseases and has been indicated as a contributor to methicillin resistance within the environment [66]. However, the horse from which the sample was taken within this current study had no reported skin health issues, although did have a mild respiratory infection [8]. Even well-established pathogens, like *S. aureus* or *S. epidermidis*, can exist in non-disease states within the skin microbiome, only causing pathology when the skin barrier is broken or when the community is disrupted [67, 68]. Thus, the detection of disease-associated genera or species on mammalian skin can provide only limited insight into host-microbe dynamics. Ultimately, more work is necessary to further characterize the interactions these microbial species might have with their mammalian hosts. Regardless, the fact that these specific *Staphylococcus* spp. have been detected on mammalian skin demonstrates the potential for *cpn60* to resolve species from vague genus classifications and for its application in universal high-resolution profiling of the mammalian skin microbiome.

## Conclusion

Mammalian skin microbial profiles generated using the *cpn60* marker gene were shown to be comparable with 16S rRNA gene datasets and supports previous 16S rRNA gene-based observations of phylosymbiosis. Evidence for phylosymbiosis was observed in the mammalian orders *Perissodactyla* and *Artiodactyla* using the *cpn60* gene amplicon dataset and phylogeny-based UniFrac distance metric; this evidence was absent in the 16S rRNA gene amplicon dataset. Resolving species from within vague genus classifications provides insight into the distribution, presence, and potential influence of specific host-associated taxa and their influence on skin health and disease. The *cpn60* gene amplicon dataset revealed previously unobserved associations between mammalian hosts and specific taxa, such as *Jeotgalicoccus halophilus*, that otherwise would have remained undetected. Amplification of the *cpn60* gene does not exclude specific bacterial communities over others (e.g., archaea-specific or species-specific primers) and can thus be used for both whole microbial community and species-specific profiling. Although the 16S rRNA gene is likely to remain as the dominantly used phylogenetic marker for amplicon-based studies, the *cpn60* gene is complementary to microbiome studies where universal low-level taxonomic resolution is desired. However, if *cpn60* amplicon studies are to be compared with those produced using the 16S rRNA gene, it is imperative that a standardized taxonomy database be maintained alongside the cpnDB sequence reference database. This study integrated cpnDB into the QIIME2 environment using the NCBI taxonomy database, allowing for more rapid analysis of *cpn60* gene amplicon datasets in the future. However, in lieu of a separate taxonomy database, progress towards integrating the cpnDB with existing taxonomy databases, such as SILVA [50, 51] or the ribosomal database project [69] would facilitate future *cpn60*-based research and improving taxonomic assignment, both within and external to the QIIME2 environment.

## Supplementary information

Figure S1

Table S1

Table S2

## Data Availability

All data generated during this current study are available in the European Nucleotide Archive (ENA) repository under project accession number PRJEB43503. The complete *cpn60* ASV table used for this study is available at https://figshare.com/articles/dataset/cpn60_ASV_table/14955753.
