# Supplementary material for: Evaluating cpn60 for high-resolution profiling of the mammalian skin microbiome and detection of phylosymbiosis
Source: ISME Commun. 2023 Jul 7;3:69. doi: 10.1038/s43705-023-00276-y (PMC10328941; doi:10.1038/s43705-023-00276-y)
Supplement: Supplementary file 1 — Figure S1 [file 43705_2023_276_MOESM1_ESM.docx]

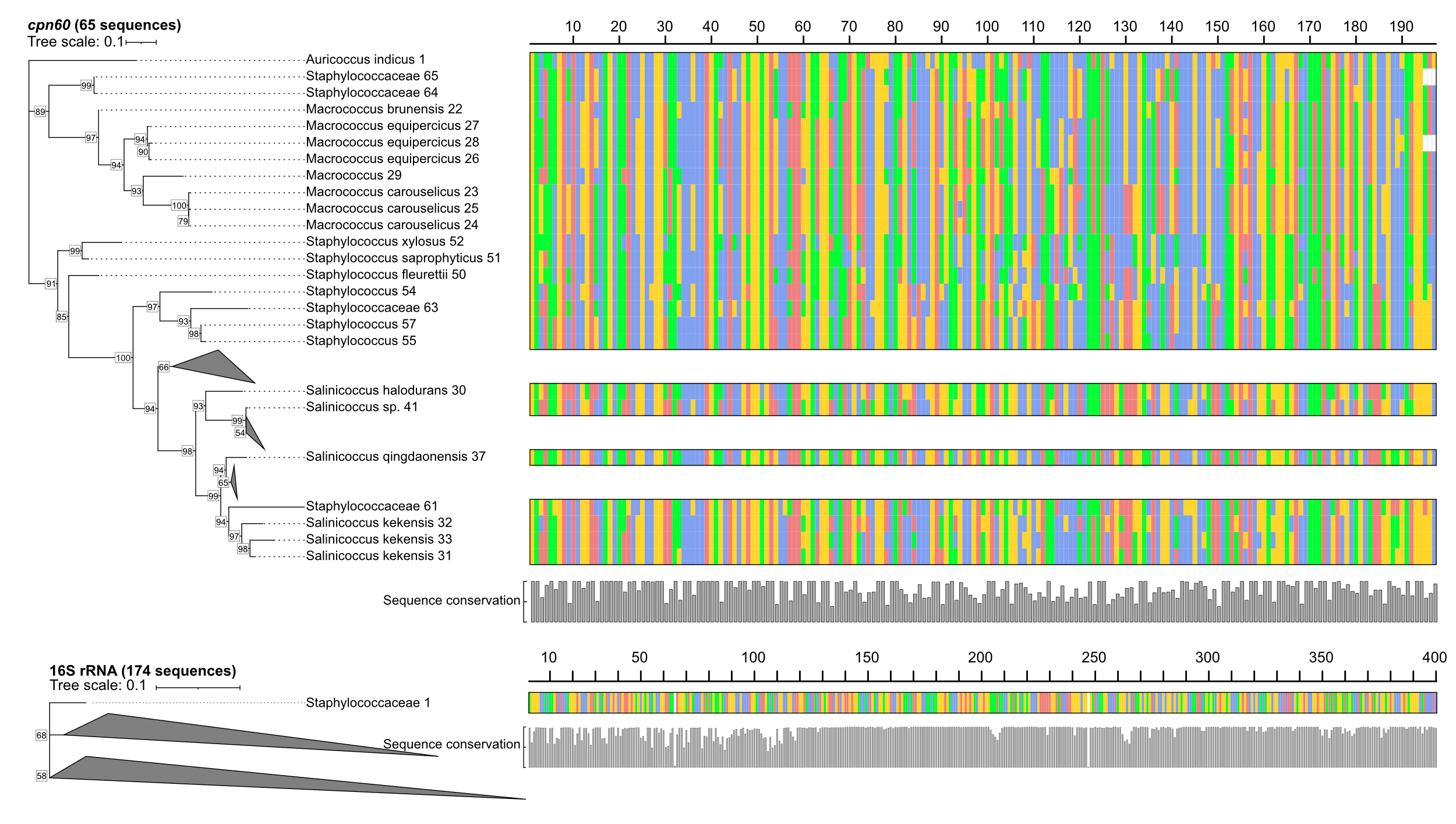


**Figure S1.** Comparison of phylogenetic resolution of family *Staphylococcaceae* between *cpn60* and 16S rRNA gene amplicon datasets. All amplicons associated with *Staphylococcaceae* from the respective datasets were aligned using ClustalW. Maximum likelihood trees were generated using the GTR +I +G model and 1000 bootstraps, and nodes with less than 75% support were collapsed. Visuals were generated using Interactive Tree of Life V5 [1].

**REFERENCES**

1. Letunic I, Bork P. Interactive Tree Of Life (iTOL) v5: an online tool for phylogenetic tree display and annotation. *Nucleic Acids Res.* 2021;**49**:W293–W296
